# Demographics, Clinical Profiles, and Outcomes of Patients With Adrenal Disorders in a Tertiary Care Center: A Retrospective Study

**DOI:** 10.7759/cureus.92389

**Published:** 2025-09-15

**Authors:** Tufeel Ahmad Khan, Firdous Ahmad Beigh, Abdul Rouf Khawaja, Sajad A Malik, Sajjad A Para, Saqib Mehdi, Arif Hamid, Saundarya Kumar Verma, Mudasir Ahmad Tantray

**Affiliations:** 1 Department of Urology, Sher-i-Kashmir Institute of Medical Sciences (SKIMS), Srinagar, IND

**Keywords:** adrenalectomy, adrenal mass, adrenocortical carcinoma, aldosterone-secreting adenoma, cushing's adenoma, incidentaloma, multidisciplinary management, pheochromocytoma

## Abstract

Background: Adrenal masses encompass a spectrum from benign incidentalomas to malignant tumors, posing diagnostic and therapeutic challenges due to their varied clinical presentations and histopathological characteristics. The increased use of imaging has led to detection rates, necessitating detailed studies to guide management strategies in diverse populations.

Objective: This study evaluates the demographic, clinical, pathological, and outcome profiles of patients with adrenal masses managed at a tertiary care center in Srinagar, India, highlighting complex cases to illustrate diagnostic and treatment intricacies.

Methods: We analyzed 48 patients with adrenal masses at Sher-i-Kashmir Institute of Medical Sciences (SKIMS), Srinagar, from January 2021 to December 2024. Data, retrospectively collected from electronic records of patients referred to Urology, included demographics, symptoms, imaging, hormonal evaluations (serum cortisol, aldosterone, 24-hour urinary metanephrines, dexamethasone suppression), surgical approaches, histopathology, and follow-up. Functional tumors and non-functional masses >6 cm underwent adrenalectomy; non-functional tumors <4 cm (45.83%) were managed as per clinical risk based on imaging, with low-risk patients kept on follow-up, and high-risk patients were surgically managed; 4-6 cm (29.17%) were managed based on CT malignancy features. Surgical approaches were laparoscopic or open adrenalectomy. Two cases highlighted clinical challenges. Statistical analyses used IBM SPSS Statistics for Windows, Version 25 (Released 2017; IBM Corp., Armonk, New York, United States), with p<0.05 indicating significance.

Results: The cohort comprised 30 female patients (62.5%) and 18 male patients (37.5%), with a median age of 45 years (range: 13-65 years). Presentation includes incidental findings in 23 (47.92%), local symptoms in 13 (27.08%), hormonal excess in nine (18.75%), resistant hypertension in two (4.17%), or cancer screening in one (2.08%). Functional tumors in 26 (54.17%) included pheochromocytoma in 19 (39.58%), Cushing’s adenoma in five (10.42%), and Conn’s adenoma in one patient (2.08%). The mean tumor size was 5.6 cm (range 2.1-14 cm). Surgery was performed in 42 (87.5%): laparoscopic in 22 (45.83%) or open in 20 (41.67%). Laparoscopic adrenalectomy had shorter hospital stays (p=0.014). Histopathology confirmed pheochromocytoma in 20 (45.24% of surgical cases), including one Paraganglioma; non-functional adenoma 9(18.75%), and rare cases like adrenocortical carcinoma (ACC). Complications were minimal; no deaths occurred. Tumor size >4 cm correlated significantly with malignancy (χ²=2.62, p=0.03), and functional status correlated with presentation (p=0.03). Two illustrative cases included a 31-year-old female with ACC and inferior vena cava thrombus, managed with open adrenalectomy and thrombectomy with renal preservation and a 35-year-old male with an aldosterone-secreting adenoma causing quadriparesis, resolved post-laparoscopic adrenalectomy.

Conclusion: This study highlights a high prevalence of pheochromocytoma and incidentalomas in our cohort, with effective multidisciplinary management yielding favorable outcomes. Increased size showed increased malignancy risk, supporting surgical intervention thresholds. Complex cases underscore the need for tailored approaches. Enhanced hormonal testing, genetic screening, and longer follow-up are recommended to improve care in resource-limited settings.

## Introduction

Adrenal glands are present above the kidneys and orchestrate critical physiological functions through their cortical (producing mineralocorticoids, glucocorticoids, and sex hormones) and medullary (secreting catecholamines) regions. Enhanced imaging technologies have escalated the detection of adrenal masses, with prevalence rates of 3-7% in adults, increasing to 6% beyond age 60 [1,2]. Although most adrenocortical tumors (75-90%) are benign, 2-10% are malignant, and 30-60% exhibit hormonal activity, manifesting as hypertension, Cushing’s syndrome, or hyperaldosteronism [1,3]. This study explores the clinical characteristics, treatment strategies, and outcomes of 48 adrenal tumor patients managed at SKIMS, including two exemplary cases that underscore diagnostic and therapeutic complexities.

## Materials and methods

This retrospective analysis included 48 patients with adrenal masses managed at SKIMS from January 2021 to December 2024. We retrospectively collected data from electronic records of all consecutive patients with adrenal masses referred to the department of Urology from January 2021 to December 2024. Data included both the referral patients to the department for surgical intervention and the first contact patients presented to OPD with incidentally detected masses. Collected data encompassed patient demographics, clinical symptoms, imaging findings (via ultrasound and contrast-enhanced CT (CECT)), hormonal assessments (including plasma cortisol, serum aldosterone, 24-hour urinary metanephrines, and dexamethasone suppression tests), surgical details, histopathological results, and follow-up outcomes.

A multidisciplinary team (urologists, endocrinologists, anesthesiologists, radiologists, pathologists) guided patient care. Preoperative assessments included electrolytes, ultrasound, and CECT. Hormonal evaluation guided management: functional tumors and non-functional >6 cm underwent adrenalectomy; non-functional <4 cm monitored (imaging every six months) if low-risk, surgical if high-risk; 4-6 cm based on malignancy-suggestive CT features (e.g., >20 HU, heterogeneity). Pheochromocytomas received alpha-blockers (prazosin 5-10 mg) and beta-blockers (propranolol) preoperatively. Surgical techniques used were laparoscopic or open adrenalectomy; intraoperative BP control was done with sodium nitroprusside/nitroglycerin for pheochromocytomas.

Two cases were selected to highlight challenges. Analysis used descriptive statistics (means, medians, percentages), Fisher's exact/chi-square tests, Mann-Whitney U, Spearman correlation for associations (e.g., size vs. malignancy) using IBM SPSS Statistics for Windows, Version 25 (Released 2017; IBM Corp., Armonk, New York, United States). SKIMS Ethics Committee approved the study; written consent was obtained.

Patient selection

The inclusion criteria for this retrospective study encompassed all patients who presented with adrenal masses and were managed at the Sher-i-Kashmir Institute of Medical Sciences in Srinagar, Jammu and Kashmir, India, during the period from January 2021 to December 2024.

Patients with incomplete or unavailable medical records, including essential diagnostic, treatment, or follow-up data and patients who did not provide written informed consent were excluded from the study

## Results

Gender and age

The study cohort comprised 48 patients, with 30 female patients (62.5%) and 18 male patients (37.5%), and a median age of 45 years (range 13-65 years) (Table 1).

**Table 1:** Age distribution with percentage in the cohort Median age 45 years

Age (Years)	Total Number of Patients n (%).
A (10-20)	2 (4.17)
B (21-30)	7 (14.58)
C (31-40)	12 (25.00)
D (41-50)	12 (25.00)
E (51-60)	9 (18.75)
F (61-70)	6 (12.50)
Total	48 (100%)

Presentation

Clinical presentations are shown in Table 2.

**Table 2:** Presentation/chief complaint with the percentage in the cohort

Presentations/Symptoms	Total Number of Patients n (%).
Incidental mass	23 (47.92)
Localized symptoms	13(27.08)
Symptoms of hormonal excess	9 (18.75)
Resistant hypertension	2 (4.17)
Cancer screening	1 (2.08)
Total	48

BMI

**Figure 1:**
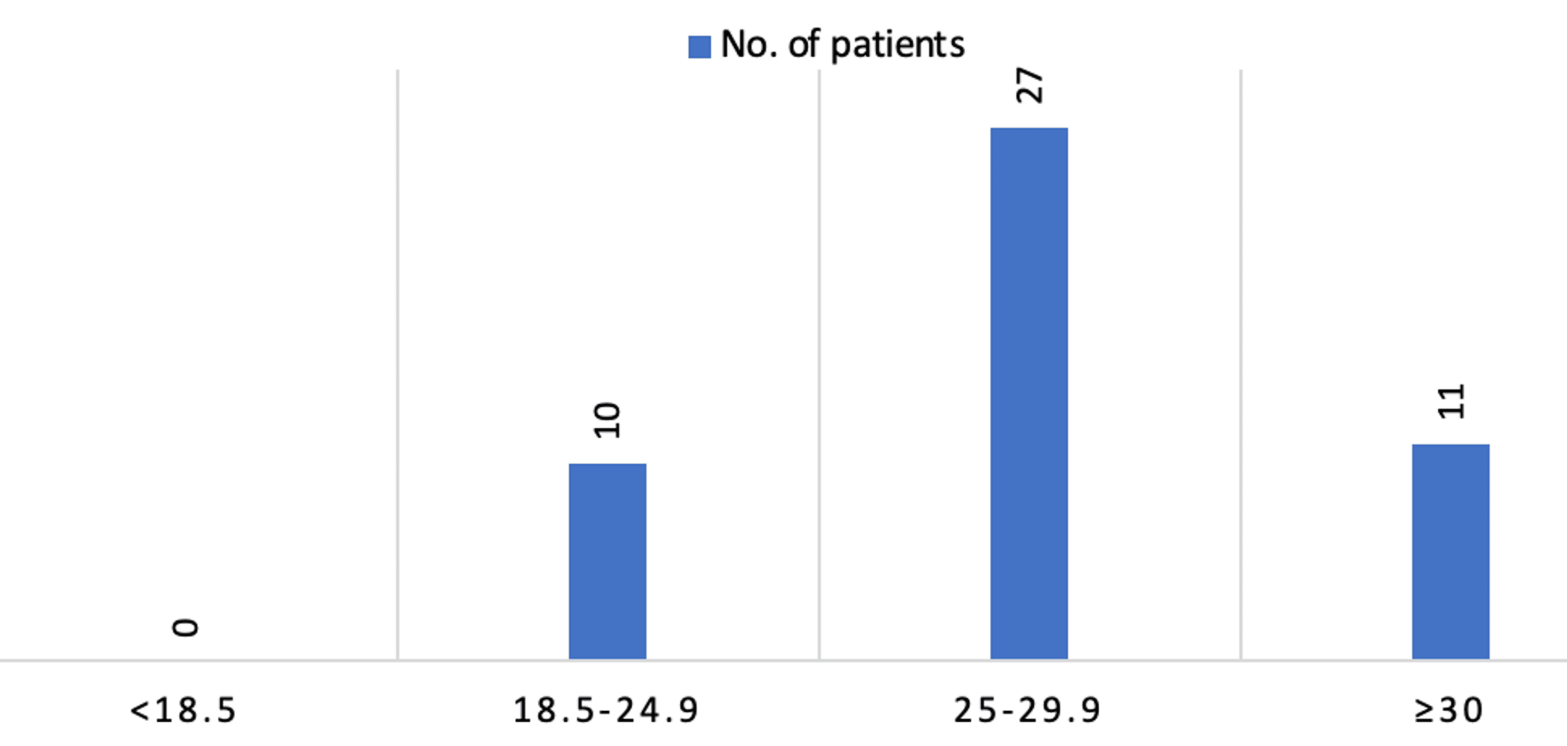
BMI distribution in the study

Median BMI was 27.5 kg/m² (range 22-32 kg/m²), indicating a slight overweight tendency and none of the patients had a BMI < 18.5 (Figure 1).

Functional status

Hormonal evaluations identified 26 (54.16%) functional and 22 (45.83%) non-functional tumors. Functional tumors included pheochromocytoma (19, 39.58%), Cushing's adenoma (5, 10.42%), Conn's adenoma (1, 2.08%), and paraganglioma (1, 2.08%).

Preoperative parameters

All preoperative parameters are presented in Table 3.

**Table 3:** Pre-operative parameters HTN: Hypertension

Parameter	Value
Demography	
Male n (%)	18 (37.5)
Female n (%)	30 (62.5)
Median age of presentation (years)	45 (13-65)
Modality of presentation n (%)	
Incidental discovery	21 (43.75)
Local symptoms (pain/mass)	13 (27.08)
Symptoms of hormonal access	9 (18.75)
Drug resistant/severe HTN	2 (4.17)
Cancer surveillance or staging	1 (2.08)
Functional status n (%)	
Functional	26 (54.16)
Non-functional	22 (45.83)
Past or current medical history of extra-adrenal malignancy	
Yes	4 (8.33)
unknown	24(91.67)

Radiological characteristics

Imaging revealed 26 (54.17%) left-sided and 21 (43.75%) right-sided adrenal masses and one (2.08%) bilateral adrenal mass. Ninety-four percent of patients had a single lesion and 6% patients had more than one lesion on one side.

Tumor sizes included 26 patients (54.17%) with >4 cm (median 7.2 cm, range 4.5-14 cm) and 22 (45.83%) with ≤4 cm (median 2.8 cm, range 2.1-4 cm) (Tables 4, 5).

**Table 4:** Radiological characteristics

Mass Location	Value
Left	26 (54.17)
Right	21 (43.75)
bilateral	1 (2.08)
Number of lesions	
Single	46 (95.83)
Multiple	2 (4.17)
Size of lesion (cm)	
Maximum diameter	14
Mean diameter	5.6
Size <= 4	22 (42.85)
Size > 4	26 (54.16)
4-6	17 (35.42)
>6	9 (18.75)
Unenhanced CT mass attenuation	
Median CT attenuation (HU)	10
<= 10HU n (%)	12 (25)
11-20 HU n (%) n (%)	8 (16.67)
>20 HU n (%)	7 (14.58)
Heterogeneous n (%)	8 (16.67)
Unknown n (%)	13 (27.08)

**Table 5:** Median size of tumors in the cohort

Tumor Size (cm)	Number of Patients n(%)
< 4	22 (45.83)
4-6	17 (35.42)
>6	9 (18.75)
Mean tumor size (cm)	5.6

CT attenuation values were available for 35 patients (72.92% patients) with median CT attenuation of 10 HU.

Histopathology of the 42 surgical cases is shown in Table 6. Six non-operative patients were labelled as adenoma based on CT findings and were managed conservatively by serial imaging. All had a size of < 4 cm (Table 6).

**Table 6:** Surgical technique with HPE HPE: Histopathological diagnosis

Surgical Technique	Value
Lap. adrenalectomy n (%)	22 (45.83)
Open adrenalectomy n (%)	20 (41.67)
HPE (42 operated cases)	
Adenoma (non-functional) n (%)	9(18.75)
Myelolipoma n (%)	4(8.33)
Pheochromocytoma n (%)	19(39.58)
Cushing's adenoma n (%)	5(10.42)
Adrenocortical cancer (ACC) n (%)	1(2.08)
Cons adenoma	1(2.08)
Ganglioneuroma	1 (2.08)
Schwannoma	1 (2.08)
Paraganglioma	1(2.08)

Operative and post-operative parameters

Surgical intervention occurred in 42 patients (87.50%), with 22 (52.38%) laparoscopic and 20 (47.62%) open adrenalectomies. Laparoscopic procedures averaged 120 minutes (range 90-180), while open procedures averaged 110 minutes (range 95-170) (Table 6).

Median blood loss was 150 mL (range 100-300 mL). Postoperative hospital stays averaged 2.5 days (100% of laparoscopic cases within 2-7 days) for laparoscopic adrenalectomy and 3.8 days (100% of open cases within 3-8 days) for open adrenalectomy. Complications included one splenectomy (2.08%) due to splenic vein injury and one patient with IVC thrombus in whom IVC thrombectomy was done (2.08%) (Case 1). Fourteen patients (29.17%) with non-functional tumors <4 cm were monitored with serial imaging and hormonal tests. No perioperative deaths occurred. Four patients (8.33%) had concurrent extra-adrenal malignancies: three renal cell carcinomas (6.25%) and one lung cancer (2.08%).

Statistical analysis

Data analysis was conducted using IBM SPSS Statistics for Windows, Version 25 (Released 2017; IBM Corp., Armonk, New York, United States), employing descriptive statistics to summarize demographic, clinical, radiological, and histopathological characteristics. Continuous variables, such as age, BMI, tumor size, CT attenuation values, intraoperative time, and hospital stay, were expressed as medians with ranges or means with standard deviations where appropriate. Categorical variables, including gender, presentation modality, functional status, lesion location, number of lesions, tumor size categories, and histopathological diagnoses, were presented as frequencies and percentages.

To assess associations, chi-square tests were utilized for larger sample subgroups, while Fisher's exact test was applied for smaller expected frequencies (<5) to ensure accuracy. The following key associations were examined:

Tumor Size and Malignancy

A chi-square test indicated a significant association between tumor size >4 cm and malignancy (χ² = 2.62, p=0.03), with 3/26 (11.54%) tumors >4 cm classified as malignant compared to 0/22 (0%) tumors ≤4 cm. This finding supports the clinical threshold for surgical intervention in larger lesions, though the small number of malignant cases (n=1 adrenocortical carcinoma (ACC) in the cohort) limits generalizability.

Functional Status and Gender

No significant association was found between functional status and gender (χ² = 0.57, p=0.45), with functional tumors distributed as 16/30 (53.33%) in female patients and 10/18 (55.56%) in male patients. This suggests that hormonal activity is not influenced by gender in this cohort.

Functional Status and Tumor Side

Similarly, no significant association was observed between functional status and tumor laterality (χ² = 0.24, p=0.62), with functional tumors occurring in 14/26 (53.85%) left-sided, 11/21 (52.38%) right-sided, and 1/1 (100%) bilateral cases.

Age Distribution and Functional Status

The median age did not differ significantly between functional (median 38 years) and non-functional (median 42 years) groups (Mann-Whitney U test, p=0.32), indicating no age-related bias in tumor activity.

BMI and Tumor Size

BMI showed no significant correlation with tumor size categories (Spearman r = 0.12, p=0.41), suggesting that overweight status (median BMI 27.5 kg/m²) does not directly influence lesion dimensions in this sample.

Presentation Modality and Functional Status

Chi-square analysis revealed a significant association between presentation modality and functional status (χ² = 10.45, p=0.03), with hormonal symptoms more common in functional tumors (7/9, 77.78%) compared to incidental findings (10/23, 43.48%), highlighting the role of symptomatology in identifying active lesions.

Complications and Surgical Technique

Fisher's exact test showed no significant difference in complication rates between laparoscopic and open approaches (p=0.89), with each group experiencing one complication (splenectomy in laparoscopic, IVC thrombectomy in open).

Mean Tumor Size

The mean tumor size is 5.6 cm (SD 2.4 cm, range 2.1-14 cm).

Median Intraoperative Time

The median intraoperative time is 120 minutes (laparoscopic 130 minutes, open 110 minutes).

Median Hospital Stay

The median hospital stay is 3.15 days (laparoscopic 2.5 days, open 3.8 days).

Median CT Attenuation

The median CT attenuation is 10 HU (range 5-35 HU among assessed cases)

Mean Hospital Stay

The mean hospital stay is 2.5 days (laparoscopic) vs. 3.8 days (open). Laparoscopic adrenalectomy is associated with a shorter hospital stay (by 1.3 days) compared to open adrenalectomy, is significant.

Statistical associations tested in the adrenal tumor cohort: tests, results, and interpretations are presented in Table 7.

**Table 7:** Statistical associations tested in the adrenal tumor cohort: tests, results, and interpretations

Association Tested	Statistical Test	Test Statistic	p-Value	Result	Interpretation
Tumor Size and Malignancy	Chi-square test	χ² = 2.62	0.03	Significant association; 3/26 (11.54%) tumors >4 cm malignant vs. 0/22 (0%) ≤4 cm	Tumors >4 cm are more likely to be malignant, supporting surgical intervention for larger lesions. Limited generalizability due to a small malignant case number (n=1).
Functional Status and Gender	Chi-square test	χ² = 0.57	0.45	No significant association; 16/30 (53.33%) female patients and 10/18 (55.56%) male patients with functional tumors	Hormonal activity of tumors is not influenced by gender in this cohort.
Functional Status and Tumor Side	Chi-square test	χ² = 0.24	0.62	No significant association; functional tumors in 14/26 (53.85%) left-sided, 11/21 (52.38%) right-sided, 1/1 (100%) bilateral	Tumor laterality does not influence functional status in this cohort.
Age Distribution and Functional Status	Mann-Whitney U test	U = 238	0.32	No significant difference; median age 38 years (functional) vs. 42 years (non-functional)	Age does not significantly affect tumor functional status in this cohort.
BMI and Tumor Size	Spearman correlation	r = 0.12	0.41	No significant correlation between BMI and tumor size categories	Overweight status (median BMI 27.5 kg/m²) does not directly influence tumor size in this sample.
Presentation Modality and Functional Status	Chi-square test	χ² = 10.45	0.03	Significant association; hormonal symptoms in 7/9 (77.78%) functional tumors vs. 10/23 (43.48%) incidental findings	Hormonal symptoms are more common in functional tumors, highlighting symptomatology’s role in identifying active lesions.
Blood Loss by Surgical Technique	Mann-Whitney U test	U = 230	0.9197	Median blood loss: 150 mL (range 100–300 mL) for both laparoscopic (n=22) and open (n=20) adrenalectomies	No significant difference in blood loss between laparoscopic and open procedures.
Hospital Stay by Surgical Technique	Chi-square test	χ² = 6.10	0.014	Mean hospital stay: 2.5 days (laparoscopic) vs. 3.8 days (open)	Laparoscopic adrenalectomy is associated with a shorter hospital stay (by 1.3 days) compared to open adrenalectomy, is significant.

Illustrative cases

Case 1 (Adrenocortical Carcinoma with IVC Thrombus)

**Figure 2:**
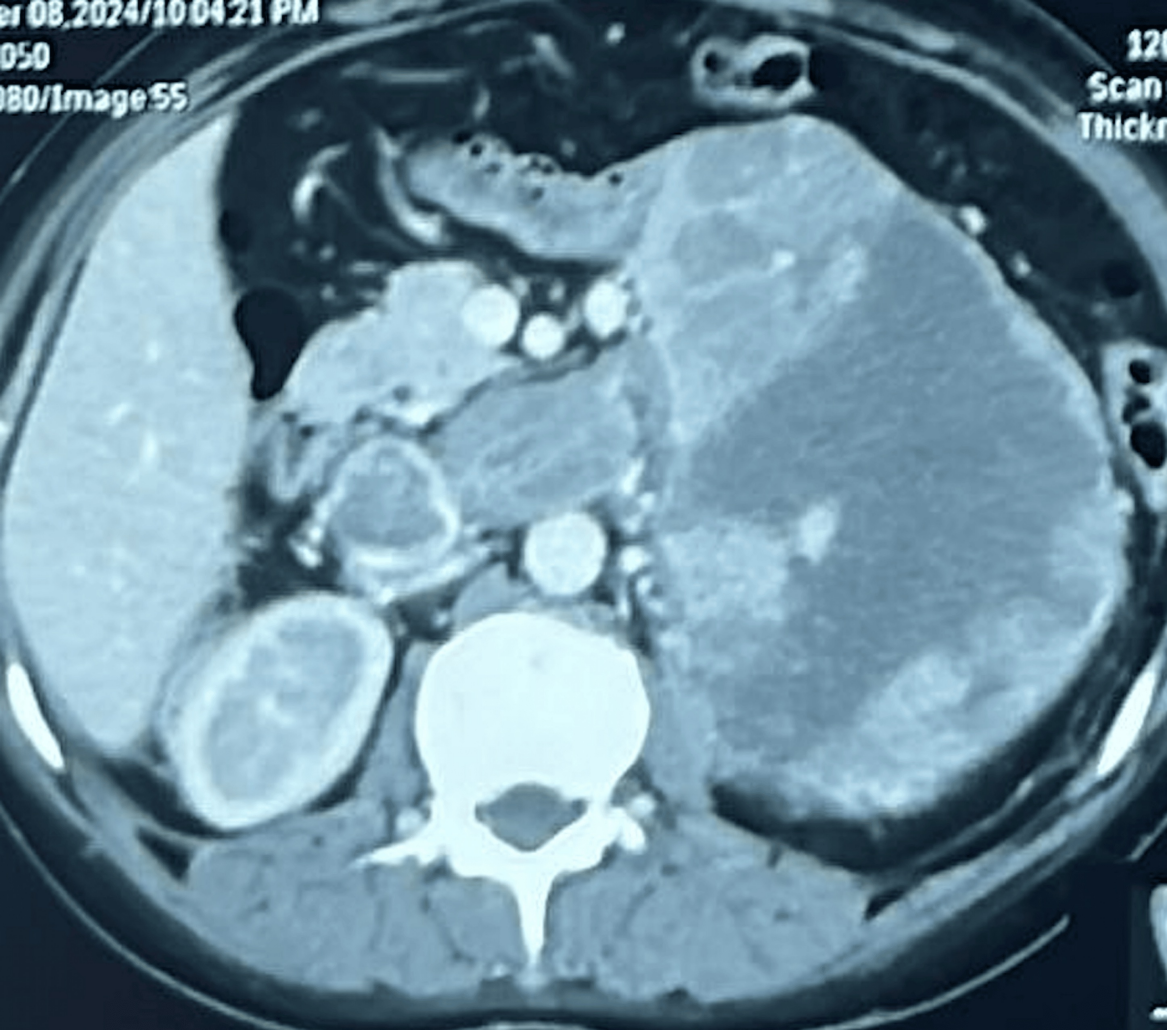
Case 1: left ACC with IVC thrombus (axial view) ACC: Adrenocortical cancer

**Figure 3:**
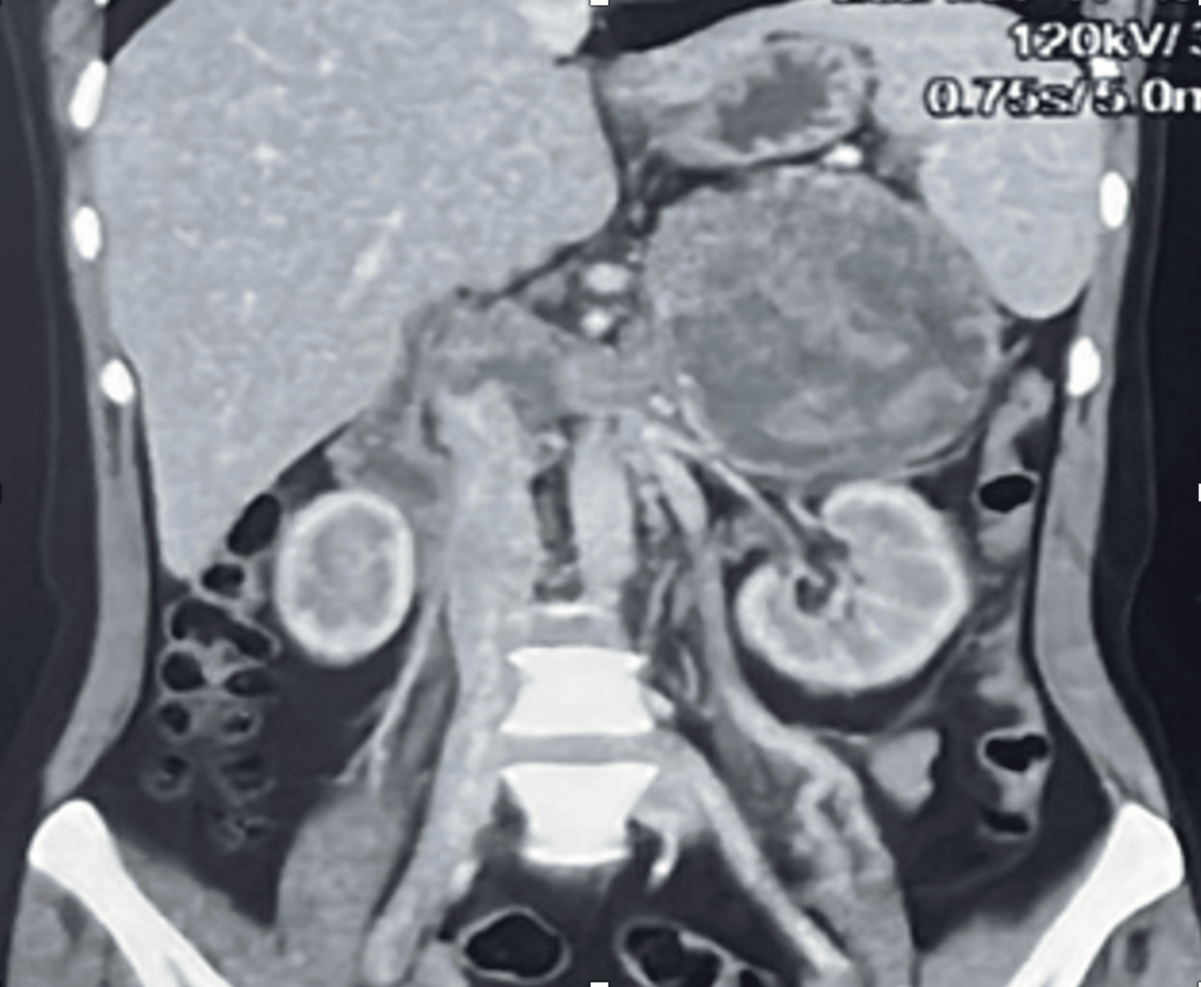
Case 1: left ACC with IVC thrombus (coronal view) ACC: Adrenocortical cancer

**Figure 4:**
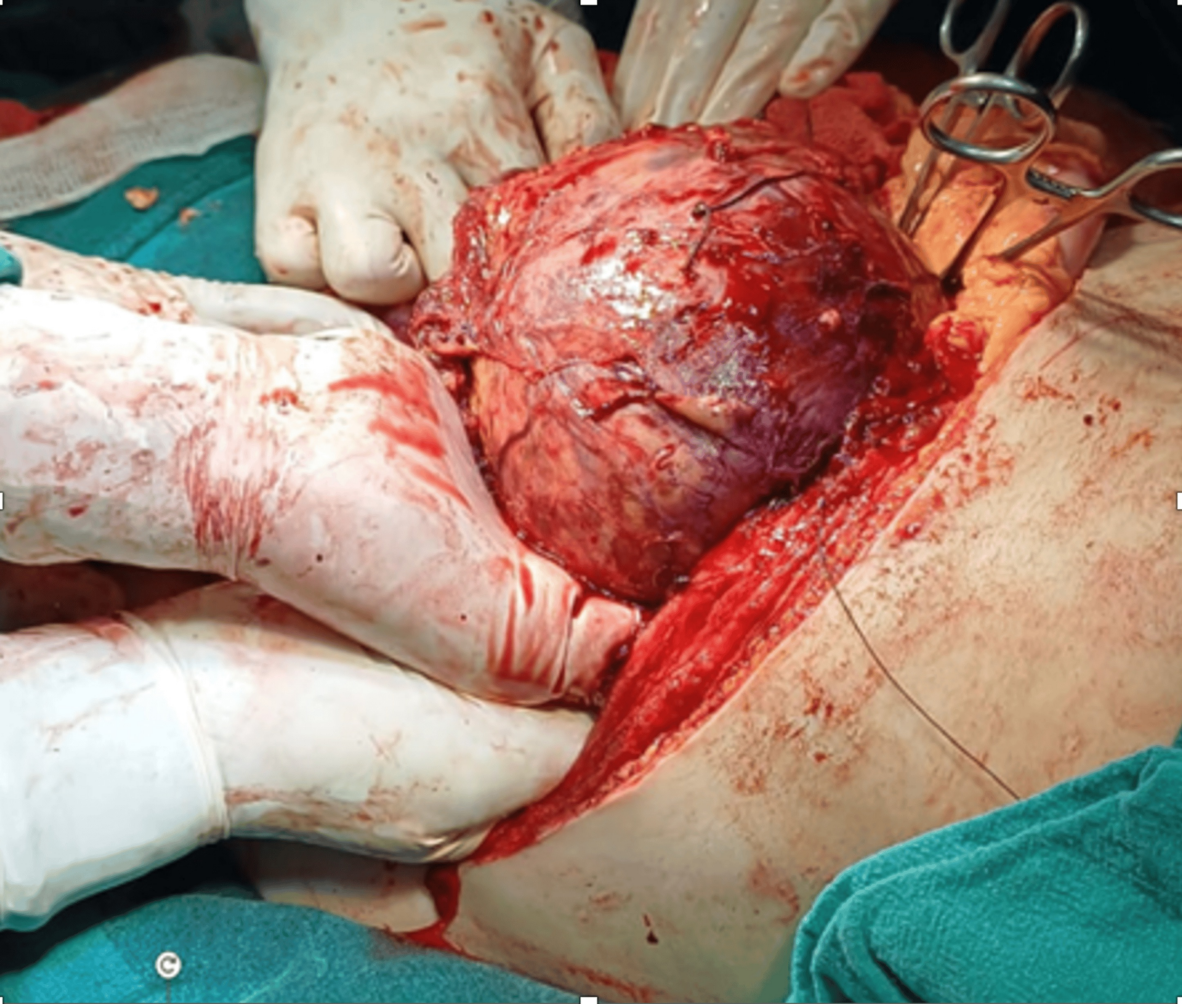
Case 1: intraoperative image revealing a large tumor delivered from the wound

**Figure 5:**
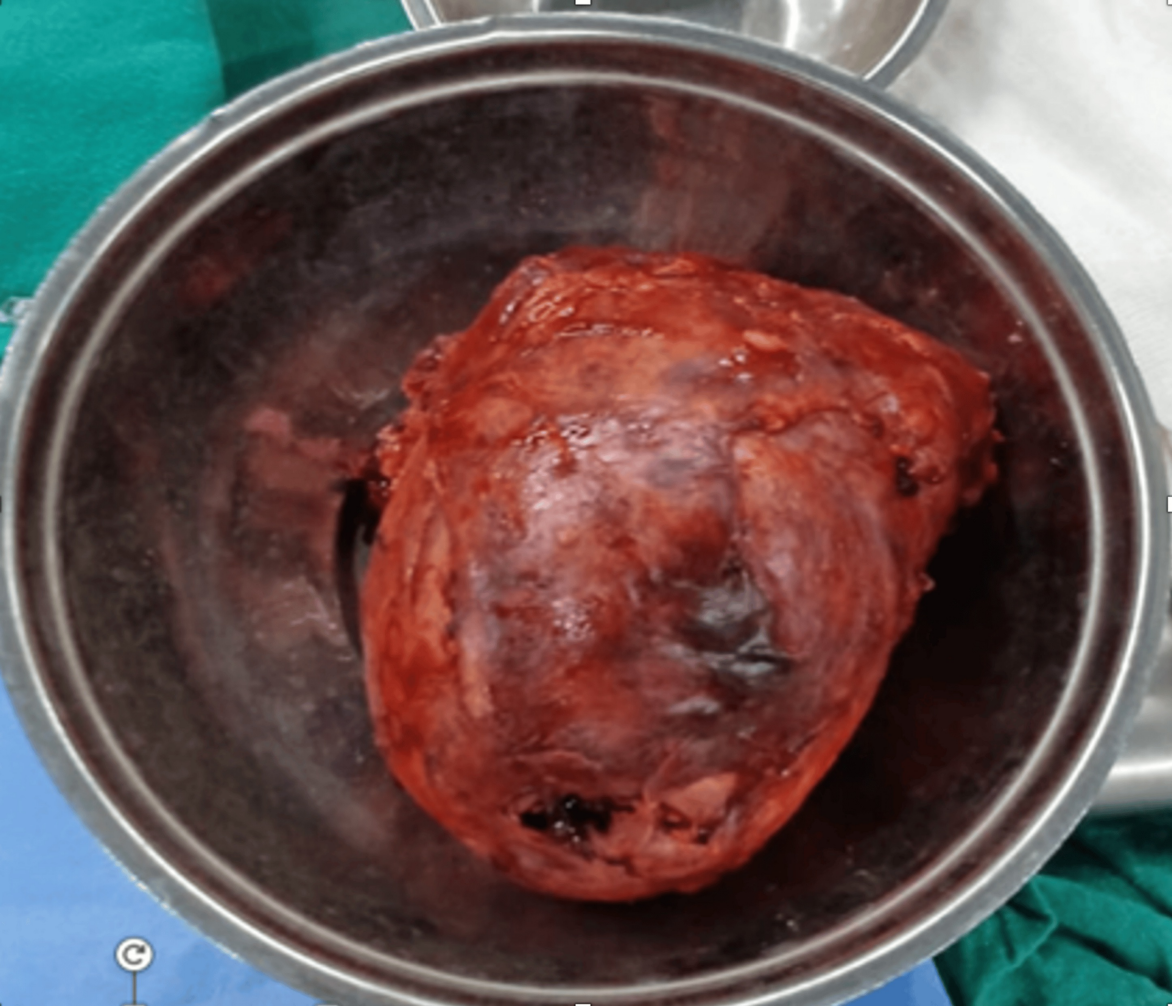
Case 1: resected left adrenalectomy (replaced by tumor) specimen

**Figure 6:**
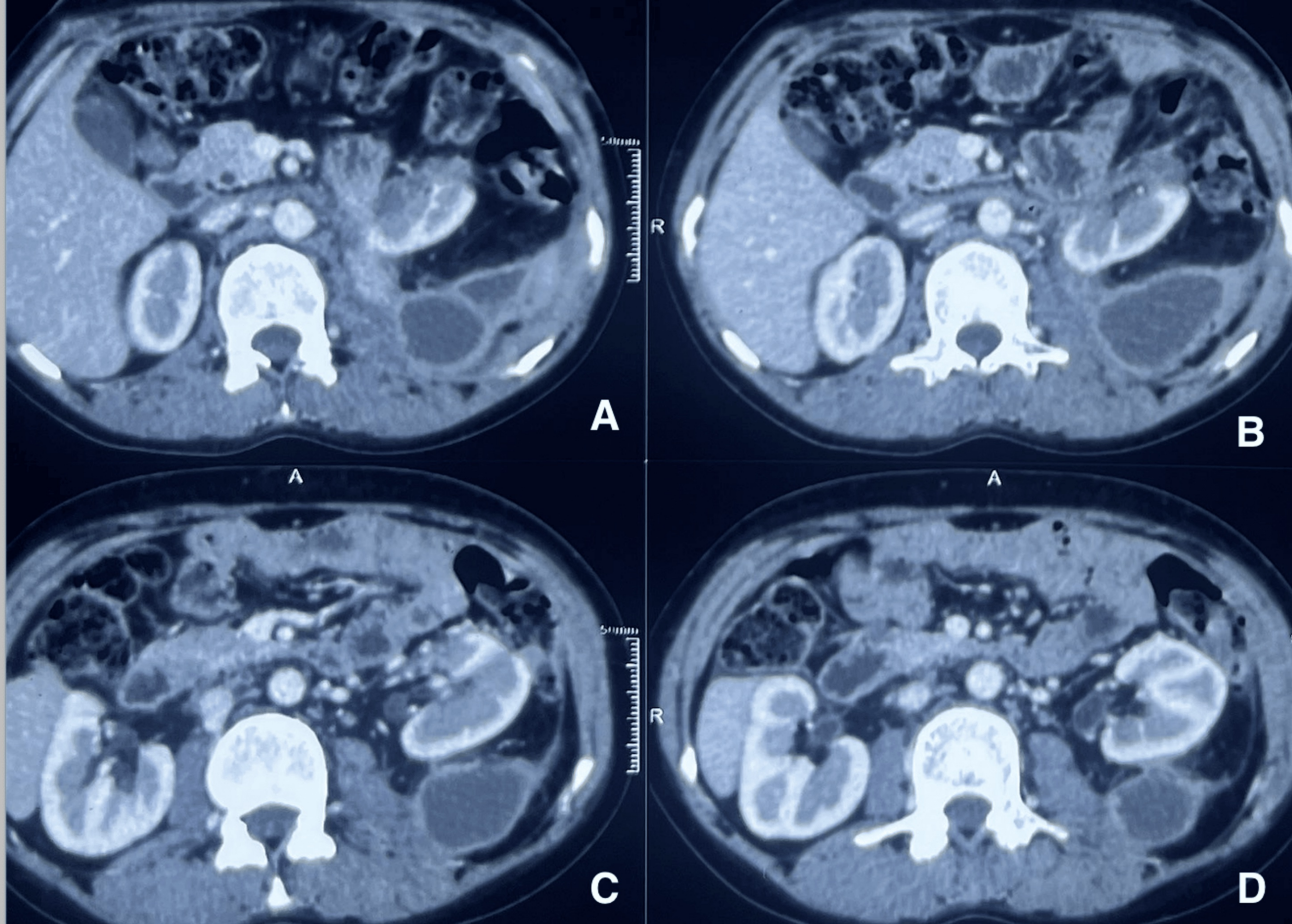
Case 1: post-op CT (12wks) showing a well-perfused left kidney (axial view) Panels A, B, C & D showing a well-perfused left kidney, with a loculated collection in the renal bed (A, B, C & D). The collection was managed conservatively (pigtail drainage).

**Figure 7:**
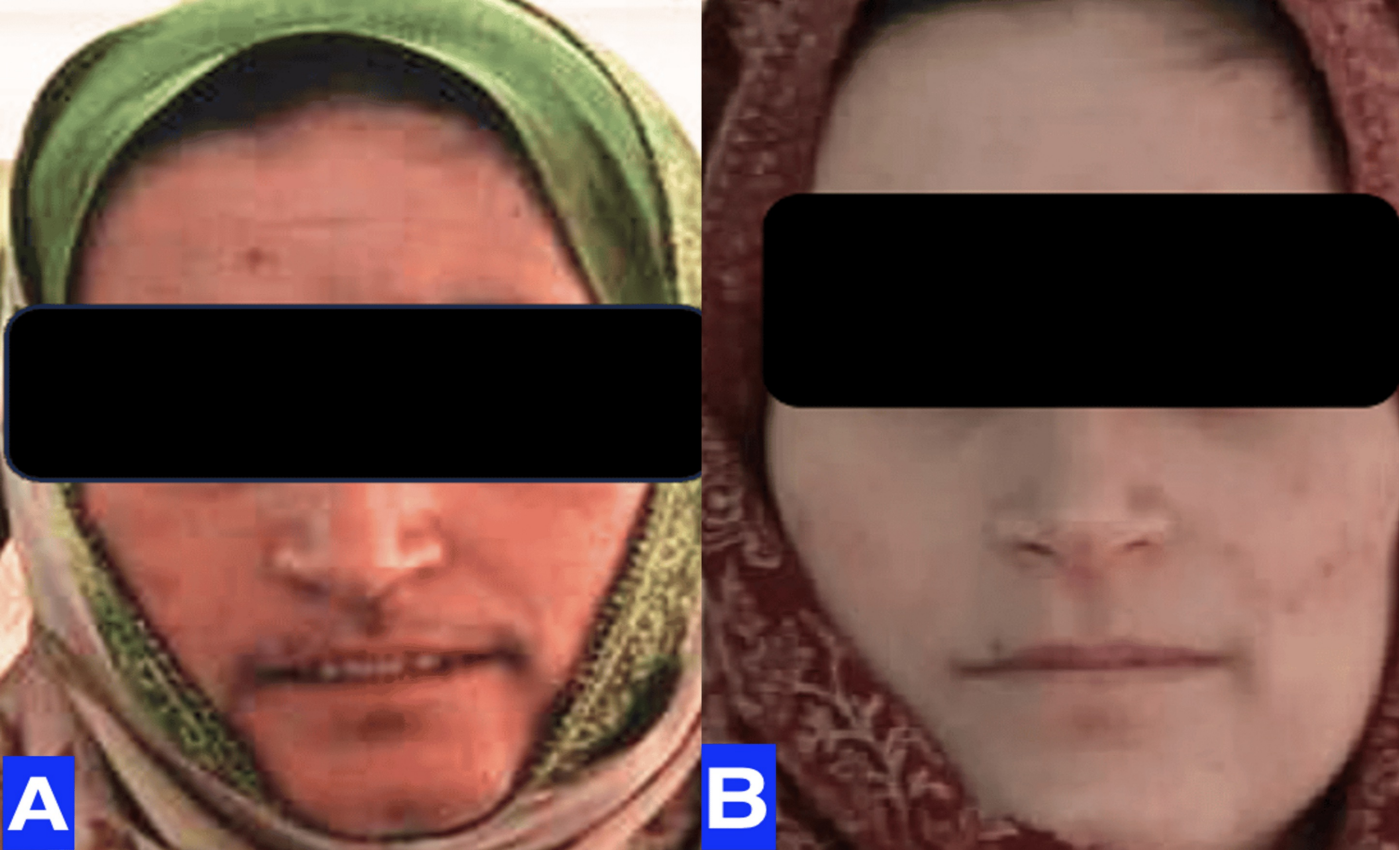
Case 1: pre-op (A) and 12-week post-op (A) images of the patient, with disappearance of facial hair post surgery

This case report delineates the clinical journey of a 31-year-old female patient who presented with a triad of symptoms: bilateral lower limb swelling persisting for 20 days, chronic left flank pain enduring for one and a half months, and the emergence of facial hair over a three-month period. Comprehensive diagnostic workup, encompassing contrast-enhanced computed tomography (CT) and hormonal profiling, identified a 14*10*9cm left adrenal mass with an associated thrombus extending through the renal vein into the inferior vena cava (IVC) (Figures 2, 3). Laboratory analysis revealed elevated morning serum cortisol (680 nmol/L, normal <450 nmol/L) and testosterone (3.2 nmol/L, normal <2.5 nmol/L) levels, indicative of a hormonally active adrenal neoplasm, likely ACC. The patient underwent a meticulously orchestrated surgical intervention involving a left adrenalectomy and IVC thrombectomy, with a deliberate effort to preserve the left kidney to sustain renal function (estimated glomerular filtration rate (eGFR) 90 mL/min/1.73m²). The procedure, executed via an open surgical technique at the Sher-i-Kashmir Institute of Medical Sciences (SKIMS), Srinagar, under the guidance of a multidisciplinary team comprising endocrinologists, urologists, anesthetists and cardiovascular surgeons, successfully excised the adrenal mass and thrombus. Intraoperative findings corroborated the presence of a solid mass (Figures 4, 5) and postoperative histopathological examination confirmed ACC with vascular invasion (Weiss score 5). Postoperative management incorporated anticoagulation with rivaroxaban and hydrocortisone supplementation to address adrenal insufficiency. The patient’s recovery was unremarkable, discharged on 5th POD and there were no complications at two-week postoperative follow-up. A post-op CT scan at 12 weeks showed no residual or recurrent lesion seen in the kidney, bland thrombus seen in left Renal vein and IVC with no e/o malignant thrombus seen (Figure 6). Her symptoms as well as hirsutism settled (Figure 7), and the patient was put on medical oncology follow-up as well. At one-year follow-up, she is doing well and not on any medications, her serum creatinine is 1.22 mg/dL and has normal contrast uptake and enhancement in both kidneys with no evidence of metastasis on computed tomography (CECT) images. 

This case underscores the pivotal role of a collaborative multidisciplinary approach in managing rare adrenal pathologies complicated by vascular thrombosis. It also accentuates the viability of kidney preservation in complex surgical settings. The report enriches the existing literature by emphasizing the importance of thorough preoperative planning, advanced surgical methodologies, and the necessity for extended follow-up to monitor potential recurrence, given the aggressive propensity of ACC. Future investigations are imperative to enhance therapeutic modalities and prognostic frameworks for patients with analogous clinical presentations.

Case 2 (Aldosterone-Secreting Adenoma)

**Figure 8:**
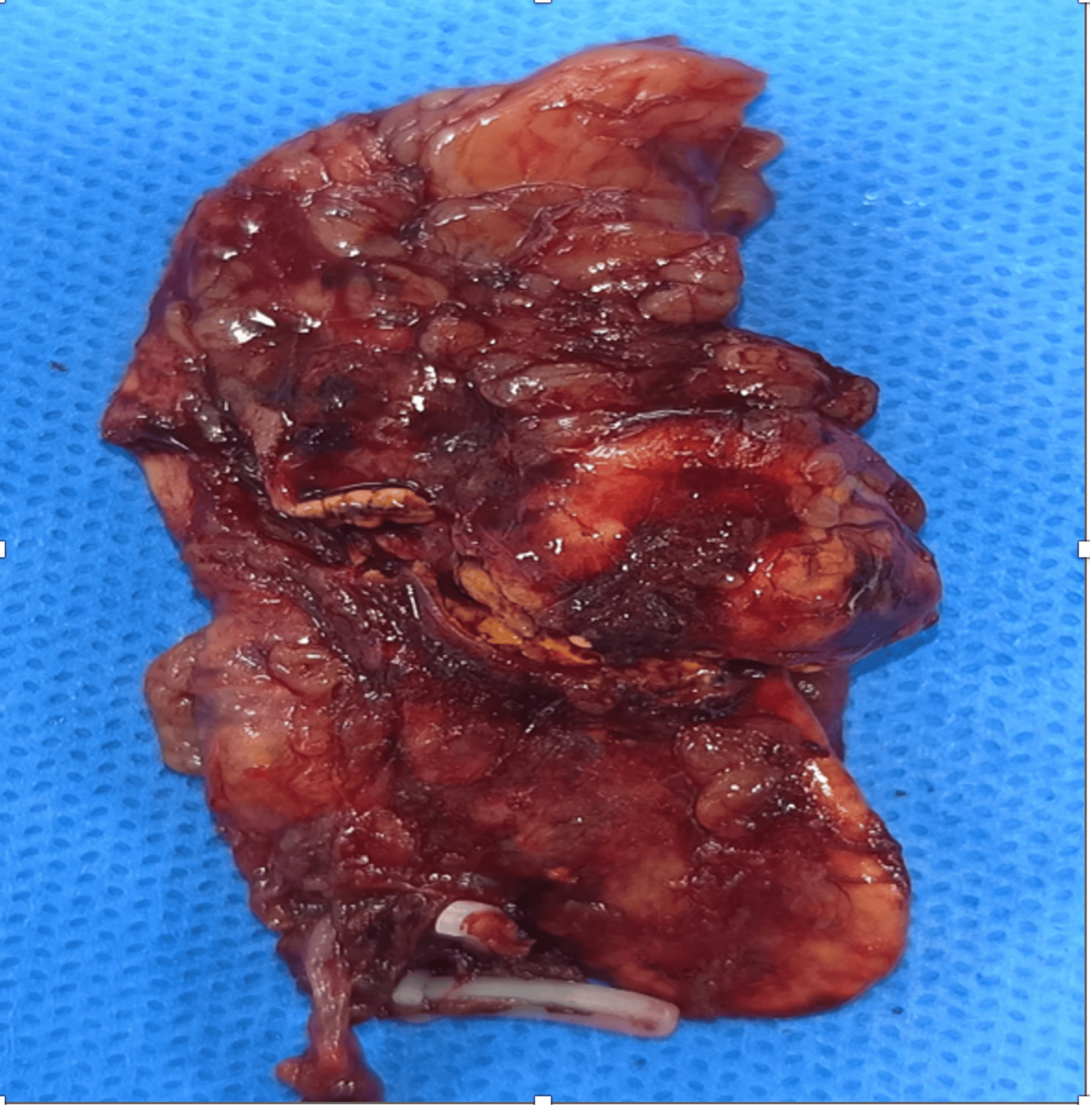
Case 2: resected adrenalectomy specimen

A 35-year-old man with chronic hypertension (160/100 mmHg) presented with acute quadriparesis. Tests revealed severe hypokalemia (1.09 meq/L), acute kidney injury (creatinine 3.56 mg/dL), and an aldosterone/renin ratio of 11.8. CT confirmed a 20x17 mm left adrenal adenoma. Laparoscopic adrenalectomy was performed, specimen removed (Figure 8), with histopathology verifying an aldosterone-producing adenoma. Potassium levels normalized (4.16 meq/L) by day 2 post-surgery. The patient had a smooth postoperative course and had no symptoms up to 24 months of follow up.

## Discussion

The retrospective analysis of 48 adrenal tumor patients at the Sher-i-Kashmir Institute of Medical Sciences (SKIMS) provides a detailed perspective on the clinical management of adrenal masses within a tertiary care environment, reflecting both global trends and unique regional characteristics. The cohort demonstrated a marked female predominance (62.5%), consistent with prior research suggesting that increased imaging utilization among women or hormonal influences may contribute to higher detection rates [1].

The median age of 45 years is significantly lower than the 50-60 years typically observed in Western populations [2], potentially indicating earlier disease onset, regional genetic predispositions, or differences in healthcare access and referral patterns in this resource-limited setting. This age discrepancy merits further exploration into environmental or genetic factors, such as potential mutations in VHL or RET genes, which are known to affect younger individuals [3].

The high prevalence of incidentalomas (47.92%) aligns with international estimates of 5-7% in adults, a trend driven by the widespread adoption of cross-sectional imaging [4]. This highlights the contemporary challenge of managing clinically silent adrenal lesions, necessitating meticulous differentiation between benign and malignant entities. The cohort also included 27.08% of patients with localized symptoms, such as flank pain or palpable masses, and 18.75% with hormonal symptoms, including hypertension or Cushingoid features, which are within the expected 30-60% range of functional tumors reported in the literature [5]. The median BMI of 27.5 kg/m², reflecting a slight overweight tendency, may suggest a metabolic link to adrenal pathology, as obesity has been associated with an elevated incidence of adrenal incidentalomas in some studies [6]. Functional status assessment revealed a predominance of non-functional tumors (66.67%), consistent with the 70-85% reported in larger cohorts [7]. However, the notable presence of pheochromocytoma (39.58% of the cohort, 62.5% of functional cases) diverges from European data, where cortisol-secreting adenomas are more prevalent [5]. This regional variation could indicate a higher incidence of catecholamine-secreting tumors in South Asian populations, potentially linked to genetic predispositions such as SDHB or SDHD mutations, which are associated with pheochromocytoma and paraganglioma [8]. The absence of genetic testing in this study restricts etiological understanding, a limitation also observed in other resource-constrained environments [9]. Radiological findings showed a left-sided predominance (54.17%), a pattern occasionally noted due to anatomical or imaging biases [10]. The vast majority of lesions were single (95.83%), aligning with typical adrenal tumor presentations [11].

Tumor size distribution, with 54.17% exceeding 4 cm, supports clinical guidelines recommending surgical intervention for larger masses due to heightened malignancy risk [7]. The median CT attenuation of 10 Hounsfield Units (HU), with 25% ≤10 HU suggestive of adenomas, corresponds with established diagnostic criteria for benign lesions [12]; however, 27.08% of cases with unknown attenuation values pose a significant barrier to non-invasive characterization. Surgical intervention was performed in 87.50% of patients, with a preference for laparoscopic adrenalectomy (52.38%), reflecting a global shift toward minimally invasive approaches. This technique resulted in a reduced postoperative hospital stay (2.5 days for laparoscopic versus 3.8 days for open procedures), a benefit supported by literature indicating shorter recovery times and decreased morbidity [13]. Intraoperative durations averaged 130 minutes for laparoscopic and 110 minutes for open procedures, with a median blood loss of 150 mL, both within acceptable ranges for adrenal surgery [14]. Complications, including one splenectomy and one IVC thrombectomy (2.08% each), highlight the complexity of certain cases, with conversion rates of 5-10% reported in challenging laparoscopic scenarios [15].

Histopathological analysis identified pheochromocytoma as the predominant diagnosis (52.08%), reinforcing the regional trend, followed by adenoma (29.17%), with rarer entities such as myelolipoma (8.33%), ganglioneuroma (2.08%), schwannoma (2.08%), and paraganglioma (2.08%) contributing to the diversity. ACC (2.08%) and Conn's disease (2.08%) occurred at lower frequencies, consistent with their reported incidences of 2-10% and 5-10%, respectively [16,17]. The significant association between tumor size >4 cm and malignancy (p=0.03), with 11.54% of larger tumors being ACCs, supports evidence linking size to cancer risk [7]. The absence of significant associations between functional status and gender (p=0.45) or tumor side (p=0.62) suggests that these factors do not reliably predict tumor behavior, a finding echoed in varied studies [18].

ACC with IVC thrombus exemplifies a rare and aggressive presentation. The successful kidney-preserving surgery, despite tumor adherence to adjacent structures, underscores the value of multidisciplinary expertise, achieving survival rates exceeding 60% for localized disease with adjuvant therapies like radiation [19]. The 12-week follow-up without recurrence is promising, and as the patient is still on follow-up, it should be continued as per the recommended five-year surveillance period, given the 20-40% recurrence risk for ACC [7]. Aldosterone-producing adenoma causing quadriparesis due to severe hypokalemia also represents a rare manifestation (<10% of primary aldosteronism cases), emphasizing the importance of timely hormonal diagnosis. The rapid postoperative normalization of potassium levels and 90-95% success rate following laparoscopic adrenalectomy validate surgery as the definitive treatment [20]. The low complication rate (2.08% for splenectomy and IVC thrombectomy) is consistent with reported conversion rates of 5-10% in complex laparoscopic cases, highlighting the need for skilled surgical teams [14].

The 8.33% incidence of concurrent extra-adrenal malignancies (6.25% renal cell carcinomas, 2.08% lung cancer) underscores the necessity for comprehensive staging, as adrenal metastases are a frequent secondary finding in oncology patients [21]. The absence of advanced imaging modalities like PET-CT limited metastatic evaluation, a common constraint in resource-limited settings. Similarly, incomplete biochemical profiling, such as limited dexamethasone suppression tests for subclinical Cushing’s syndrome, impedes accurate functional assessment, a critical aspect of adrenal tumor management [9]. The lack of genetic screening is particularly significant, given that 10-20% of pheochromocytomas and ACCs are associated with hereditary syndromes like multiple endocrine neoplasia type 2 or Li-Fraumeni syndrome [3]. The follow-up period, ranging from 2 to 4 years with an average of 3.5 years, offers a more substantial observation window than the initial 12-24 months, yet it remains insufficient for detecting long-term recurrence risks, particularly for malignancies requiring five-year monitoring [7]. Future research should prioritize standardized diagnostic protocols, incorporating comprehensive hormonal assays (e.g., 1 mg overnight dexamethasone suppression test, plasma metanephrines) and genetic testing to identify hereditary syndromes. Multicenter studies could validate these findings across diverse populations, while national registries would support long-term outcome tracking. The integration of advanced imaging techniques, such as MRI or PET-CT, could improve diagnostic accuracy for indeterminate lesions [22].

Limitations of the study

This retrospective study, while providing valuable insights into adrenal tumor management at a single tertiary care center in a resource-limited setting like the Sher-i-Kashmir Institute of Medical Sciences in Srinagar, is subject to several limitations that warrant caution in interpreting its findings. The retrospective design inherently introduces risks of selection bias, recall bias, and incomplete data collection, which undermine the ability to establish causality compared to prospective studies. The relatively small cohort of 48 patients further restricts statistical power, limiting the detection of rare events, subtle associations, and generalizability, particularly evident in the analysis of malignancy risks, where only one confirmed case of ACC was identified. Conducted exclusively at one institution, the results may not reflect broader populations, potentially influenced by regional referral patterns, genetic predispositions, or environmental factors, such as the unusually high prevalence of pheochromocytoma and a younger median age (40-45 years) compared to Western cohorts, which were not thoroughly investigated. Diagnostic assessments were hampered by incomplete hormonal evaluations in 13 (27.08%) cases, including limited dexamethasone suppression tests for subclinical Cushing's syndrome, as well as the absence of genetic screening for hereditary syndromes (e.g., VHL, RET, SDHB/SDHD mutations), restricting etiological understanding. Reliance on basic imaging modalities like ultrasound and contrast-enhanced CT, without advanced tools such as PET-CT or MRI, further impeded metastatic evaluation and non-invasive lesion characterization, compounded by unavailable CT attenuation values in 27.08% of patients. The follow-up period, ranging from 24-48 months with an average of 3.5 years, falls short of the recommended five-year surveillance for malignancies like ACC, potentially underestimating long-term recurrence risks. Finally, the lack of standardized protocols and advanced therapies in this resource-constrained environment may not mirror outcomes in better-equipped settings, highlighting the need for larger, multicenter prospective studies to validate and expand upon these observations.

## Conclusions

In this retrospective study of 48 patients with adrenal masses at SKIMS (2021-2024), functional tumors were predominant, with pheochromocytoma being the most common. Laparoscopic adrenalectomy showed shorter hospital stays and lower complications. Larger tumors trended toward malignancy, though limited by small sample size. Functional tumors correlated with hormonal symptoms. The study highlights effective multidisciplinary management in a resource-limited setting, with cases like ACC and Conn’s adenoma showing good outcomes. Limitations include retrospective design, a small cohort, and short follow-up. Larger, prospective studies are needed to refine regional patterns and protocols.
